# Feedback-Related Electroencephalogram Oscillations of Athletes With High and Low Sports Anxiety

**DOI:** 10.3389/fpsyg.2018.01420

**Published:** 2018-08-28

**Authors:** Hiroaki Masaki, Takahiro Hirao, Yuya Maruo, Dan Foti, Greg Hajcak

**Affiliations:** ^1^Faculty of Sport Sciences, Waseda University, Saitama, Japan; ^2^Department of Physical Education, Tokyo Women’s College of Physical Education, Tokyo, Japan; ^3^Department of Psychological Sciences, Purdue University, West Lafayette, IN, United States; ^4^Department of Psychology, Florida State University, Tallahassee, FL, United States

**Keywords:** performance monitoring, delta oscillation, feedback-related negativity, sports anxiety, choking under pressure

## Abstract

We investigated the relationship between performance-related anxiety and the neural response to error feedback that was delivered during the execution of a time estimation task. Using the Sport Anxiety Scale (SAS-2), we selected university athletes high and low in sports anxiety. Participants executed a time estimation task where they were instructed to estimate 1 s by pressing a button after a sound cue. They performed this task while their performance was being evaluated by an experimenter (evaluation condition) and also while alone (in a no-evaluation condition). We tested whether feedback-related brain activities may increase in amplitude in the evaluation condition compared to the control condition – especially for athletes who report high performance-related anxiety. We focused on oscillations of sub-delta, delta, and theta frequency bands phase-locked to the feedback onset. Time-frequency analyses revealed that the magnitude of both the sub-delta component (0.3–1.2 Hz) and the theta component (4–8 Hz) were larger in incorrect than correct trials. In addition, the theta component was smaller for athletes high in sports anxiety than for athletes low in sports anxiety. The delta component was overall larger for correct than incorrect feedback. Further, athletes high in sports anxiety exhibited a larger delta component (1.5–3.5 Hz) for correct feedback in the evaluation condition than in the no-evaluation condition. Our results suggest that evaluation by others may increase the delta oscillation associated with correct feedback processing – especially among athletes high in sports anxiety.

## Introduction

Sport behavior is a dynamic and efficient form of action. Although elite athletes exhibit enhanced and sophisticated movements during sporting competitions, some are prone to choke under pressure (i.e., perform worse than usual) due to anxiety that occurs preceding or during an important competition (Baumeister, 1984). Indeed, we do not understand exactly why some elite athletes choke under performance pressure–and little is known about the neural basis of pressure-induced performance decrements, an understanding which might help develop improved coping techniques.

One previous study suggests that neural correlates of performance monitoring may differ among athletes who are prone to choking under pressure (Masaki et al., 2017). Masaki and colleagues focused on an event-related potential (ERP) referred to as the error-related negativity (ERN) (Falkenstein, 1990; Gehring et al., 1990). The ERN is obtained by averaging electroencephalogram (EEG) synchronized to the onset of erroneous responses, and reflects a generic neural system that implements performance monitoring (e.g., Ullsperger and von Cramon, 2001). The ERN peaks within 100 ms following the erroneous response over frontocentral regions, and is thought to be generated in the anterior cingulate cortex (ACC) (Dehaene et al., 1994). Previous studies have shown that the ERN amplitude is modulated through attentional control (e.g., Gehring et al., 1993; Tanaka et al., 2005; Ogawa et al., 2011) as well as anxious traits (Hajcak et al., 2003). Masaki et al. (2017) found that the ERN was increased among athletes high in sports anxiety when performance of a spatial Stroop task was being evaluated by an experimenter, whereas it was not increased by performance evaluation for athletes low in sports anxiety. These results were in line with previous findings that the ERN was larger when subjects’ performance was being evaluated by others (Hajcak et al., 2005). Thus, sports anxious athletes may show higher ACC sensitivity to their errors during evaluation or pressure compared to when they are not being evaluated.

In tasks such as the spatial Stroop, participants are aware when they make mistakes. However, in some tasks, participants must utilize performance feedback to determine whether a response was correct. Previous studies have confirmed that processing of external feedback can be examined by the feedback-related negativity (FRN) that is elicited by incorrect feedback (Miltner et al., 1997). In the current study, we sought to investigate whether feedback processing is influenced by performance evaluation in anxious athletes. To our knowledge, no study has tested the relationship between feedback-related processing and sports anxiety that may relate to choking under pressure.

Given that the ERN and the FRN share apparent similar function and may rely on common ACC activity (Miltner et al., 1997; Gehring and Willoughby, 2002; Holroyd and Coles, 2002), it is reasonable to presume that the feedback-related brain activities would increase for individuals high in performance-related anxiety in an evaluation situation. Thus, we predicted that the feedback-related brain activities that include the FRN as well as other feedback-elicited positivities would be enhanced during the evaluation condition compared to the no-evaluation condition for athletes who are prone to choke under pressure.

In terms of frequency-band characteristics of feedback-related neural activity, theta frequency is increased following negative feedback, whereas the delta frequency band is increased following positive feedback (Bernat et al., 2008, 2011; Nelson et al., 2011; Foti et al., 2015). To examine feedback-related processing, we focused on oscillations of delta and theta frequency phase-locked to feedback onset, because these phase-locked oscillations can isolate error- and correct-related cognitive activities from other activities (Yordanova and Kolev, 2004; Yordanova et al., 2004). In this study, we applied time-frequency (TF) analysis to feedback-elicited EEG by focusing on three different frequency bands (i.e., 4–8 Hz theta, 1.5–3.5 Hz delta, and 0.3–1.2 Hz sub-delta) according to a previous study (Yordanova and Kolev, 2004). This allowed us to investigate individual components of feedback-related activities consisting of FRN in the theta frequency band, and both P300 and reward positivity (RewP) (Baker and Holroyd, 2011; Proudfit, 2015) in the delta frequency band, and slow wave or the late positive potential (LPP) that is modulated by outcome valence (Pornpattananangkul and Nusslock, 2015; Donaldson et al., 2016) in the sub-delta frequency band. We predicted that activity in these frequency bands would all be increased during evaluation compared to no evaluation condition for athletes who are prone to choke under pressure (i.e., both correct and incorrect feedback to be more salient among athletes high in sports anxiety).

To this end, we selected university athletes high in sports anxiety and low in sports anxiety using the Sport Anxiety Scale (SAS-2). All participants completed a time estimation task wherein participants estimate 1 s by a button press and receive feedback after each trial; this task was completed while performance was being evaluated by an experimenter and also during a control (i.e., no evaluation) condition. Thus, we tested whether individual frequency bands (i.e., theta, delta, and sub-delta) would be enhanced for athletes high in sports anxiety, as a function of performance evaluation.

## Materials and Methods

### Participants

Two hundred sixteen undergraduate athletes from the Faculty of Sport Sciences in Waseda University completed the Sport Anxiety Scale (SAS-2), which assesses the competitive trait anxiety of athletes. We invited those students who scored above 34 (higher sport anxiety) and below 20 (lower sport anxiety) to participate in a laboratory experiment^1^. These criteria were determined on the basis of ± 1 standard deviation (SD) from the mean total SAS-2 score (*M*: 27.2, *SD*: 7.1). Consequently, 16 individuals with higher sports anxiety (7 females, mean age ±*SD* = 20.6 ± 1.26; SAS-2 range: 34–52) and 16 with lower sports anxiety (8 females, mean age ±*SD* = 21.1 ± 2.28; SAS-2 range: 15–20) participated in this study. A *t*-test comparing SAS-2 scores between individuals with higher (*M*: 37.4, *SEM*: 1.08) and lower sport anxiety (*M*: 17.4, *SEM*: 0.43) confirmed the difference between the two groups [*t*(30) = 17.2, *p* < 0.0001, *d* = 6.09].

Participants had normal or corrected-to-normal vision and were paid 3,200 yen (about 26 USD) for their participation. All participants gave written informed consent prior to the experiment. This study was approved by the Waseda University Ethics Committee.

### Time Estimation Task

**FIGURE 1:**
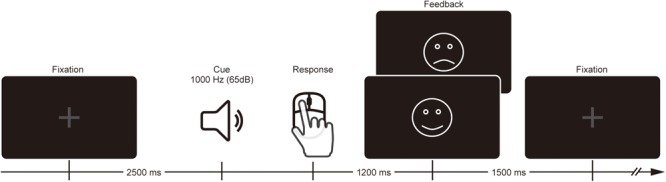
Schematic illustration of the procedure.

Participants performed a time estimation task where they were instructed to estimate 1 s by pressing a mouse button after the presentation of the sound cue (1,000 Hz, 65 dB, 200 ms duration) (**Figure 1**). Each trial began with a gray fixation cross that remained on the screen until feedback presentation. The sound cue was presented 2,500 ms after the onset of the fixation cross. Participants were asked to press the button with the right index finger whenever they thought 1 s elapsed following the sound cue. On each trial, visual feedback was either a smiley face (for correct response) or sad face (for incorrect response), and was presented at the center of the monitor to inform the participant of the outcome 1,200 ms after their estimation response. The visual angle and duration of the feedback was 3.8° × 3.6°and 1,500 ms, respectively. Estimation within the correct time range (i.e., 900–1,100 ms) at the beginning of the task was defined as a correct response. The correct range was expanded 20 ms when the previous trial was incorrect or shortened 20 ms when the previous trial was correct, which produced a total correct response rate of ∼50%. Although participants were paid for their participation, neither monetary reward nor punishment was contingent upon their individual performance.

Participants performed the task in two conditions. In the evaluation condition, the research assistant was behind the participant and pretended to evaluate the performance of the participant. In the no-evaluation condition, participants were left alone in a room to perform the task alone. Each condition consisted of two blocks (30 trials/block). The order of conditions was counter-balanced across participants using either ABBA or BAAB sequence.

### Physiological Recordings

We recorded the electroencephalogram (EEG) from 128 scalp sites according to the Biosemi electrode coordinates (Biosemi Inc., Amsterdam, Netherlands). Participants wore a nylon mesh cap, in which Ag/AgCl electrodes were embedded. We also recorded the horizontal electrooculograms (HEOG) from the left and right outer canthi and the vertical electrooculograms (VEOG) from above and below the left eye. All these physiological indices were recorded with a bandwidth of DC to 205 Hz using the Biosemi Active Two system (Biosemi Inc., Amsterdam, Netherlands), which amplifies EEG signals via active electrodes. Both EEG and EOGs were digitized at a rate of 1,024 Hz and offsets were kept below 20, which is consistent with other studies using the Biosemi system.

### Data Analysis

#### Event-Related Potential Analysis

Off-line preprocessing of EEG data was performed using the Brain Vision Analyzer 2 (Brain Products, Gilching, Germany). The EEG was re-referenced to the average activity from all 128 scalp electrodes. After down-sampling to 256 Hz, long epochs of 8,000 ms total (i.e., from 4,000 ms prior to and 4,000 ms after feedback onset) were extracted. For ERP averaging, the EEG was band-pass filtered with 0.1–30 Hz (roll-off: 24 dB/octave). Ocular-movement artifacts were corrected using the algorithm described by Gratton et al. (1983) and epochs in which EEG variations exceeded ± 75 μV were rejected from further analysis. Segmented EEG data were averaged, pooling four frontocentral electrodes (C11, C22, C23, and C24 Biosemi coordinates) including FCz. Baselines were corrected using a the time window of 100 ms prior to the feedback onset. The ERPs were measured as the mean amplitude within the time window of 100 ms, from 230 to 330 ms after the feedback onset.

#### Time-Frequency Analysis

For the TF analysis, the same procedures as the FRN analysis was conducted except that the EEG was band-pass filtered with 0.01–30 Hz (roll-off: 24 dB/octave). After the ocular-correction and artifact-rejection, segmented EEG data were exported for the follow-up TF analysis that was conducted using MATLAB R2012a (MathWorks, Inc., Natick, MA, United States). Based on Yordanova and Kolev (2004), we focused on three different frequency bands (sub-delta: 0.3–1.2 Hz, delta: 1.5–3.5 Hz, and theta: 4.0–8.0 Hz) using the continuous Morlet wavelet transform with a resolution of 0.1 Hz, ranging from 0.1 to 13 Hz. The TF analysis was applied to the averaged current source density (CSD) segments (Yordanova and Kolev, 2004) at the FCz of each participant. Mean amplitudes in a time window of 100 ms prior to the feedback onset were used as a baseline. TF components of theta bands were measured as mean amplitudes within the time window of 100 ms between a time point of 230 and 330 ms after the feedback onset. This time window corresponded to when the FRN was elicited in the current study. The TF activity of the sub-delta band was measured as the mean amplitude within the time window of 250 ms, from 200 to 450 ms after the feedback onset. The TF activity of the delta band was measured as the mean amplitude within time window of 200 ms, from 130 to 330 ms after the feedback onset.

The mean amplitudes of ERPs and each TF component were subjected to a mixed three-way analysis of variance (ANOVA) with the between-subjects factor of group (high/low in sports anxiety) and within-subjects factors of condition (evaluation/no-evaluation) and correctness (correct/incorrect feedback).

## Results

### Performance

The mean correct rate was 54% (SEM = 1.9) in the evaluation condition and 53% (SEM = 1.5) in the no-evaluation condition for athletes high in sport anxiety, and 54% (SEM = 1.2) in the evaluation condition and 55% (SEM = 1.3) in the no-evaluation condition for athletes low in sport anxiety. As expected, the correct rate was approximately 50% for both groups. It did not differ between the evaluation and the no-evaluation conditions for either group (*t*’s ≤ 0.92, *p*’s ≥ 0.37, *d*’s ≤ 0.24). No interaction was found [*F*(1,30) = 1.06, *p* = 0.31, $\eta_{p}^{2}$ = 0.03].

### Feedback-Related Brain Activities

#### Event-Related Potential

**FIGURE 2:**
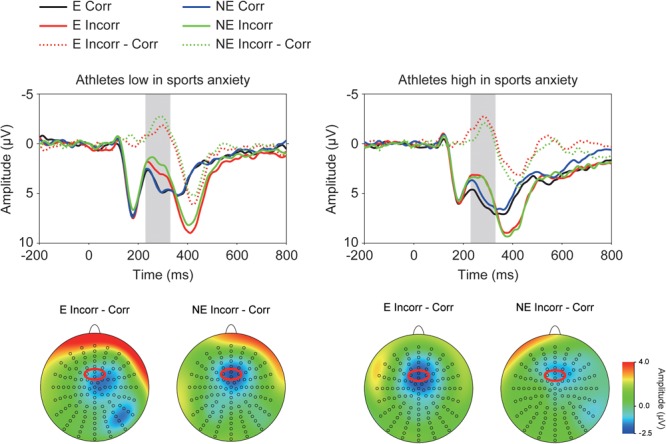
Grand-averaged waveforms of the feedback-elicited potentials for athletes high and low in sports anxiety and their topographies. The waveforms were derived from frontocentral regions that are indicated by a red circle (pooled electrodes) on the topographical maps. E: the evaluation condition, NE: the no-evaluation condition, Corr.: correct feedback, and Incorr.: incorrect feedback.

**FIGURE 3:**
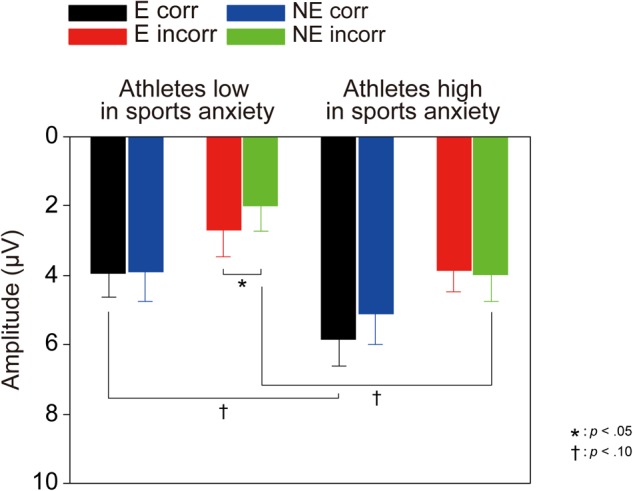
Mean amplitudes of ERPs elicited by feedback signals. Error bars represent SEM.

**Figure 2** depicts ERP waveforms of the feedback-elicited potentials, showing larger negative deflections (i.e., FRN) at approximately 250 ms following incorrect feedback. A three-way ANOVA confirmed larger negative deflections for incorrect feedback than for correct feedback [*F*(1,30) = 71.15, *p* < 0.001, $\eta_{p}^{2}$ = 0.70]. Furthermore, a three-way interaction of Group × Correctness × Condition was significant [*F*(1,30) = 4.58, *p* = 0.041, $\eta_{p}^{2}$ = 0.13] (**Figure 3**). *Post-hoc* tests showed that FRN amplitude for incorrect feedback was more negative in the no-evaluation condition than in the evaluation condition among athletes low in sport anxiety [*t*(15) = 2.22, *p* = 0.042, *d* = 0.24]. It did not differ between two conditions among athletes high in sport anxiety [*t*(15) = 0.25, *p* = 0.81, *d* = 0.04]. In the evaluation condition, ERP amplitudes for correct feedback tended to be more negative for athletes low in sports anxiety than those high in sports anxiety [*t*(30) = 1.83, *p* = 0.076, *d* = 0.65]. In the no-evaluation condition, FRN amplitudes for incorrect feedback tended to be more negative for athletes low in sports anxiety than those high in sports anxiety [*t*(30) = 1.88, *p* = 0.070, *d* = 0.66].

#### Current Source Density Derivation

**FIGURE 4:**
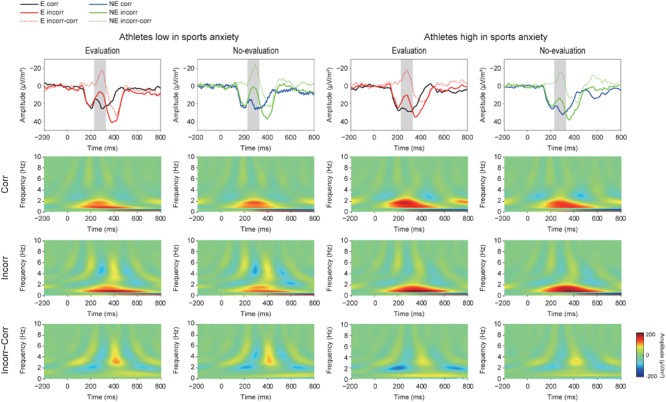
Grand averaged Current Source Density (CSD) waveforms (upper panel) (E: the evaluation condition, NE: the no-evaluation condition, Corr.: correct feedback, and Incorr.: incorrect feedback). In the CSD waveforms, black and red lines represent correct and incorrect trials in the evaluation condition, respectively. Blue and green lines represent correct and incorrect trials in the no-evaluation condition, respectively. Lower panels show Time-Frequency plots of feedback-locked activities. The two left columns represent athletes low in sport anxiety and the two right columns represent athletes high in sports anxiety.

**Figure 4** (upper panel) illustrates CSD waveforms of the feedback-elicited potentials at FCz. The CSD transform was separately conducted prior to TF analysis. We applied the same three-way ANOVA to the CSD data as was done to the FRN. It only revealed a main effect of correctness, indicating larger negative deflections for incorrect feedback than for correct feedback [*F*(1,30) = 33.09, *p* < 0.001, $\eta_{p}^{2}$ = 0.52]. Neither the main effect of condition nor the interaction between condition and group was significant.

#### Time-Frequency Analysis

**Figure 4** (lower panel) shows TF representations of the feedback-locked activities and suggest stronger activity in both the theta and sub-delta frequencies for incorrect feedback than for correct feedback, as well as increased delta frequency for correct feedback than for incorrect feedback. The TF plots of the feedback-locked activities also show larger activities of the theta frequency band (4–8 Hz) for incorrect feedback especially among athletes with low anxiety.

**FIGURE 5:**
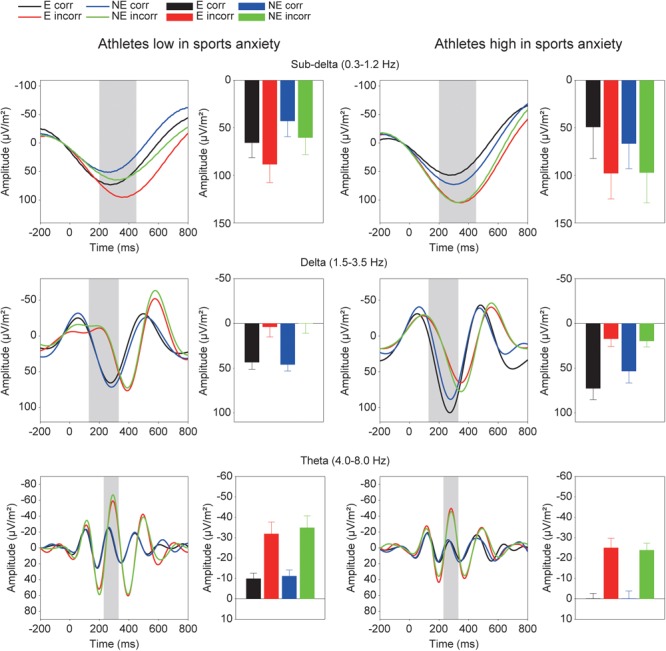
Time-frequency components (sub-delta, delta, and theta) and mean amplitudes of these components in each condition. The two left columns represent athletes low in sport anxiety and the two right columns represent athletes high in sport anxiety (E: the evaluation condition, NE: the no-evaluation condition, Corr.: correct feedback, and Incorr.: incorrect feedback).

**Figure 5** shows the grand averaged TF components of each frequency band at FCz. The sub-delta frequency band appears to represent the slow wave or the LPP that emerged about 300 ms after the feedback onset. A three-way ANOVA revealed a significant main effect of correctness, confirming larger slow waves for incorrect feedback than for correct feedback [*F*(1,30) = 9.86, *p* = 0.004, $\eta_{p}^{2}$ = 0.25]. Neither main effect of condition [*F*(1,30) = 0.78, *p* = 0.39, $\eta_{p}^{2}$ = 0.03] nor group difference [*F*(1,30) = 0.19, *p* = 0.67, $\eta_{p}^{2}$ = 0.01] was found. No interactions were found (*F’s* < 1.05).

**FIGURE 6:**
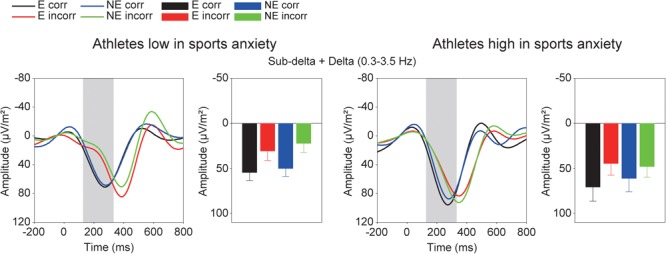
Time-frequency component (0.3–3.5 Hz). See details in Footnote 2.

For the delta frequency band, a three-way ANOVA revealed that delta activation was larger for correct than incorrect feedback signal [*F*(1,30) = 58.85, *p* < 0.001, $\eta_{p}^{2}$ = 0.66]. Furthermore, the three-way interaction of Group × Correctness × Condition was marginally significant [*F*(1,30) = 2.97, *p* = 0.095, $\eta_{p}^{2}$ = 0.09]. *Post-hoc* tests revealed that athletes high in sports anxiety had increased delta band activity for correct feedback in the evaluation compared to no-evaluation condition [*t*(15) = 2.28, *p* = 0.038, *d* = 0.37], and tended to show a more positive delta band activity for correct feedback in the evaluation condition compared to athletes low in sports anxiety [*t*(30) = 1.95, *p* = 0.061, *d* = 0.69].

For the theta frequency band, both groups showed larger oscillations for incorrect than correct feedback signal that was supported by a main effect of correctness [*F*(1,30) = 54.04, *p* < 0.0001, $\eta_{p}^{2}$ = 0.64]. In addition, the amplitude of the oscillation was larger among athletes low in sports anxiety than those high in sports anxiety [*F*(1,30) = 4.83, *p* = 0.036, $\eta_{p}^{2}$ = 0.14].^2^

## Discussion

We examined the relationship between sports anxiety and neural responses to feedback signals in a time estimation task while performance was being evaluated and in a control condition. Across these conditions, we compared feedback-locked oscillations as well as ERP waveforms between athletes with high and low sports anxiety. Although ERP amplitudes were not modulated by evaluation in athletes with high sports anxiety, their decomposed delta band component for correct feedback was enhanced by evaluation. On the other hand, both the sub-delta and the theta frequency bands were associated with the correct/incorrect status (i.e., enhancement for incorrect feedback), but not associated with evaluation. For the theta frequency band, the amplitude was also larger among athletes low in sports anxiety than those high in sports anxiety.

Previous studies of the ERN found that performance evaluation potentiated the ERN among more socially anxious participants (Hajcak et al., 2005; Barker et al., 2015; Masaki et al., 2017). Contrary to our prediction, the FRN was not potentiated by evaluation in our study. Instead, a reduced FRN amplitude was observed in the evaluation condition for athletes with low sports anxiety. According to a recent assertion, both the ERN and FRN may increase in amplitude, reflecting the demands of attentional control (van Noordt et al., 2015, 2016, 2017). Thus, it is plausible that athletes who demonstrate low levels of sports anxiety were better able to avoid distraction in the evaluation condition resulting in a reduced FRN amplitude whereas highly anxious athletes failed to efficiently control their attention to feedback under evaluation.

The null effect of evaluation on the FRN among athletes with high sports anxiety might be due to the different tasks adopted in ERN and FRN studies. Masaki et al. (2017) used a spatial Stroop task where participants monitored their own responses that relied on inhibitory control in incongruent trials to avoid errors. The participants did not require a skilled motor control for the button press, because the task demands primarily relied on temporal perception. Furthermore, feedback information about outcomes was more important to improve performance in the current study.

Beilock and Carr (2001) asserted that attentional control may shift from external to internal attention while performance is being evaluated, impairing automaticity of movements. Thus, the inward attention is thought to be responsible for choking under pressure. In the time estimation task, however, the participants might have paid attention to feedback instead of attending inward even while their performance was being evaluated, because they had to know outcomes via external feedback. It is plausible that the difference in attentional control resulted in the null effect of evaluation on the FRN in the time estimation task.

On the other hand, the TF analysis provided perspectives different from the ERP results and a more direct way to isolate individual brain activities compared to traditional ERP methods. Athletes high in sports anxiety showed increased power in the delta frequency band (i.e., 1.5–3.5 Hz) for correct feedback than incorrect feedback in the evaluation condition. Contrary to our predictions, the impact of evaluation on feedback-related brain activities only affected the delta frequency band. In terms of differentiation of function, processing of incorrect feedback was represented in both the sub-delta (i.e., 0.3–1.2 Hz) and theta (i.e., 4–8 Hz) frequency bands; both components were enhanced for incorrect compared to correct feedback. Thus, it should be emphasized that athletes high in sports anxiety showed an evaluation effect for correct feedback only in the delta frequency band, but not in other frequency bands. In addition, analyses combining the delta and sub-delta frequency bands did not show any evaluation effect, suggesting that the error-specific sub-delta band activity may have obfuscated the impact of evaluation. According to previous studies (Foti et al., 2015; Proudfit, 2015), it is reasonable to assume that the delta frequency band may include both P300 and RewP. As Proudfit (2015) proposed, the RewP may contribute to the feedback-related potentials over frontocentral regions more than P300. The RewP is attenuated (Foti and Hajcak, 2009; Foti et al., 2015) or even eliminated (Foti et al., 2011; Proudfit, 2015) when the outcome is bad, and as a result the FRN emerges. Although monetary reward was not contingent upon correct performance in our study, it is possible that success in the task provided participants with intrinsic reward especially in the evaluation condition, which might have elicited the RewP. Therefore, the enhanced delta activity in the evaluation condition might be a characteristic of athletes with high sports anxiety– they may be more sensitive to positive feedback under performance evaluation. A previous study of the ERN suggested that frequent presentation of feedback may have helped participants feel at ease, eliminating the relationship between anxiety and the ERN amplitude (Olvet and Hajcak, 2009). On the other hand, athletes low in sports anxiety did not show any enhanced delta activities for correct feedback, irrespective of evaluation. It is likely that neural response to correct feedback is not enhanced for athletes low in anxiety, because correct outcomes might not be an intrinsic reward for them.

It is well known that P300 amplitude represents the amount of attentional resources allocated to a given stimulus (Johnson, 1986). Given that the delta frequency band also includes P300 (Bernat et al., 2008, 2011; Foti et al., 2015), the present results suggest that athletes high in sports anxiety allocated attentional resources more to correct feedback in the evaluation than no-evaluation condition. This suggests that observation by others may exclusively influence feedback processing among athletes with high anxiety. However, the attentional resource might have been equally allocated to both correct and incorrect feedback processing among athletes low in sports anxiety.

Using TF analyses, Yordanova and Kolev (2004) extracted error-specific oscillations in the 1.5–3.5 Hz frequency window (delta) from multiple frequency components. They also found that the theta frequency oscillation occurred both for error and correct responses. They referred to the error-specific delta oscillation as performance monitoring system and referred to the theta oscillation as the movement monitoring system. Rather, we found correct-specific delta oscillations, that we did find error-specific sub-delta oscillation. This is not consistent with the assertion of Yordanova and Kolev (2004) that the sub-delta oscillation merely represents movement-related activities. On the other hand, we also found similar activities of the feedback-locked theta oscillation even when movement monitoring was not needed (i.e., feedback processing). That is, theta oscillation was enhanced for incorrect feedback. Thus, the feedback-locked theta frequency band may represent a more general process of outcome processing than movement monitoring.

We should also point out a limitation in our study. The three-way interaction among Group, Correctness, and Condition was marginally significant, probably due to the small sample size. Thus, further research is needed to test the delta frequency band in a larger sample of participants in order to rule out the possibility of Type I error.

Finally, in accordance with a previous report (Masaki et al., 2017), we also found that athletes low in sports anxiety were characterized by larger neural activities in the theta band than athletes with high sports anxiety irrespective of evaluation; The theta oscillations were larger for athletes low in sports anxiety than for those high in sports anxiety. These suggest that athletes with low levels of anxiety may have a distinct performance monitoring system that properly detects erroneous events. Given that lower sports anxiety results in better performance during a stressful sporting game, athletes low in sports anxiety might also have an advantage in motor skill acquisition that is consistent with a recent notion of performance monitoring during motor learning (Masaki and Sommer, 2012).

In sum, in terms of error processing, both the sub-delta and the theta frequency bands may differentiate incorrect and correct feedback activities. We found an evaluation effect on correct-feedback processing in the delta frequency band among athletes high in sports anxiety, although we did not find any evaluation effect in ERPs. This led us to conclude that decomposition of the feedback-locked activities using the TF analysis is a powerful method to reveal a small effect that is embedded in ERPs.

## Author Contributions

GH and HM designed the experiments. GH, YM, DF, and HM performed the experiments. TH and HM analyzed the data. HM, GH, YM, DF, and TH interpreted the data and wrote the paper. All authors have made direct contribution to the work and approved it for publication.

## Conflict of Interest Statement

The authors declare that the research was conducted in the absence of any commercial or financial relationships that could be construed as a potential conflict of interest.

## Footnotes

1 We also recorded ERNs of these participants in a spatial Stroop task and reported the results in Masaki et al. (2017). It should be noted that participants tested in this study were not identical to the previous report (4 participants were excluded in Masaki et al., 2017 because of fewer response errors and artifact-contaminated trials).

2 We conducted an additional analysis in the same way as other studies (e.g., Foti et al., 2015; Proudfit, 2015) where the sub-delta frequency band (0.3–1.2 Hz) was combined with the delta frequency band. **Figure 6** shows the delta activity (<3.5 Hz) without decomposition. Mean amplitudes of the delta frequency band were scored within a time window of 200 ms (ranging from 130 to 330 ms following the feedback). A 3-way ANOVA revealed that the amplitude of the delta frequency band was increased for correct response [*F*(1,30) = 43.36, *p* < 0.0001, $\eta_{p}^{2}$= 0.59]. However, neither an interaction of Correctness × Group [*F*(1,30) = 0.81, *p* = 0.37, $\eta_{p}^{2}$ = 0.03] nor an interaction of Correctness × Group × Condition [*F*(1,30) = 1.96, *p* = 0.17, $\eta_{p}^{2}$ = 0.06] was obtained.
